# Patterns and predictors of malaria care-seeking, diagnostic testing, and artemisinin-based combination therapy for children under five with fever in Northern Nigeria: a cross-sectional study

**DOI:** 10.1186/1475-2875-13-447

**Published:** 2014-11-21

**Authors:** Kathryn R Millar, Jennifer McCutcheon, Eugenie H Coakley, William Brieger, Mohammed A Ibrahim, Zainab Mohammed, Amos Bassi, William Sambisa

**Affiliations:** JSI Research & Training Institute, Inc, 44 Farnsworth Street, Boston, MA 02210 USA; Jhpiego, Baltimore, MD USA; Targeted States High Impact Project (TSHIP), JSI Research & Training Institute, Inc, Bauchi, Sokoto, Nigeria

**Keywords:** Malaria, Care-seeking, Children under five, Nigeria, Artemisinin-based combination therapy, Diagnostic test

## Abstract

**Background:**

Despite recent improvements in malaria prevention strategies, malaria case management remains a weakness in Northern Nigeria, which is underserved and suffers the country’s highest rates of under-five child mortality. Understanding malaria care-seeking patterns and comparing case management outcomes to World Health Organization (WHO) and Nigeria’s National Malaria Control Programme (NMCP) guidelines are necessary to identify where policy and programmatic strategies should focus to prevent malaria mortality and morbidity.

**Methods:**

A cross-sectional survey based on lot quality assurance sampling was used to collect data on malaria care-seeking for children under five with fever in the last two weeks throughout Sokoto and Bauchi States. The survey assessed if the child received NMCP/WHO recommended case management: prompt treatment, a diagnostic blood test, and artemisinin-based combination therapy (ACT). Deviations from this pathway and location of treatment were also assessed. Lastly, logistic regression was used to assess predictors of seeking treatment.

**Results:**

Overall, 76.7% of children were brought to treatment—45.5% to a patent medicine vendor and 43.8% to a health facility. Of children brought to treatment, 61.5% sought treatment promptly, but only 9.8% received a diagnostic blood test and 7.2% received a prompt ACT. When assessing adherence to the complete case management pathway, only 1.0% of children received NMCP/WHO recommended treatment. When compared to other treatment locations, health facilities provided the greatest proportion of children with NMCP/WHO recommended treatment. Lastly, children 7–59 months old were at 1.74 (p = 0.003) greater odds of receiving treatment than children ≤6 months.

**Conclusions:**

Northern Nigeria’s coverage rates of NMCP/WHO standard malaria case management for children under five with fever fall short of the NMCP goal of 80% coverage by 2010 and universal coverage thereafter. Given the ability to treat a child with malaria differs greatly between treatment locations, policy and logistics planning should address the shortages of essential malaria supplies in recommended and frequently accessed treatment locations. Particular emphasis should be placed on integrating the private sector into standardized care and educating caregivers on the necessity for testing before treatment and the availability of free ACT in public health facilities for uncomplicated malaria.

## Background

Malaria is a parasitic illness that affects millions around the world each year. The World Health Organization (WHO) estimates that in 2012 there were 207 million malaria cases and 627,000 malaria deaths globally; 77% of these were in children under five years of age [1]. The majority of malaria deaths are amongst children in Africa, where malaria kills one child every minute [2]. In fact, malaria accounts for 7% of deaths in children under five globally [3].

Nigeria is particularly afflicted by this disease. Malaria is the leading cause of child mortality in the nation and accounts for 30% of hospitalizations, 60% of outpatient visits [4], and 30% of deaths among children under five [5]. In 2008, there were about 100 million suspected cases and 300,000 deaths due to malaria in Nigerian children under five [6].

When prevention fails, effective malaria case management is key for preventing morbidity and mortality for children under five. Three priority areas of malaria case management—as identified by the WHO in 2010 [7] and adopted by the Nigeria National Malaria Control Programme (NMCP) [8]—are (1) prompt care-seeking within the first 24 hours of symptoms; (2) performance of a parasitological blood test, by either microscopy or rapid diagnostic testing (RDT), to prevent over- and under-diagnosis of malaria; and (3) administration of artemisinin-based combination therapy (ACT) if the blood test is positive [7]. ACT has been designated as the first-line treatment for its ability to reduce drug resistance and its efficacy in treating malaria [7]. Prompt, accurate diagnosis and treatment of malaria is essential to prevent death since the majority of malaria deaths occur within the first 24 hours following onset of fever [5].

In Nigeria, ACT was introduced as first-line treatment in 2005 and RDTs were recommended for diagnosis in 2007. The NMCP strategy for 2009–2013 [9] set a goal target of 80% of malaria cases to be diagnosed with a blood test and treated with an ACT within the first 24 hours of fever onset [8] by 2010 and universal coverage thereafter, based on goals set by RBM [6].

Despite sound policies, goals, and improvements in prevention through increased coverage of insecticide-treated nets (ITNs) [10, 11], malaria case management has continued to be inadequate in Nigeria due to weak health systems [12, 13]. As of 2013, in Nigeria, 70.1% of children under five with fever sought advice or treatment, only 11.1% received a blood test, 6.0% received an ACT and 4.2% received an ACT within 24 hours of fever onset [11]. Also, 30.9% of Nigerian children who sought treatment for malaria received chloroquine [11], a common anti-malarial, deemed ineffective in Nigeria due to resistance since the 1980s [14]. When stratified by geopolitical zone (GPZ), treatment rates were lowest in the North West and North East GPZs with only 6.7% and 8.7% receiving a blood test and 3.5% and 2.0% receiving an ACT within 24 hours of fever onset, respectively [11].

While regional data are helpful in identifying disparities generally, there is limited state-level data on malaria case management, or treatment, and patient care-seeking patterns. The 2013 Demographic and Health Survey for Nigeria details treatment received by state, but does not detail where children seek care and the treatment they receive there, by state [11]. However, this information is important and can help determine if FMOH goal coverage rates/NMCP strategies are being met, and inform policy and programme implementation by state MOHs, NGOs, and other organizations to improve case management [15]. Given the high burden of disease and poor health systems in these regions, this information is especially important.

To help address this paucity of information, a cross-sectional study was conducted in two states in Northern Nigeria. The primary aim of the study was to describe the current care-seeking and treatment pattern for children under five with fever in Northern Nigeria. The secondary aims were to determine how many children with fever receive treatment consistent with NMCP/WHO standards and which factors help predict if a child under five with fever is taken to treatment, none of which have been studied in Nigeria to date.

## Methods

### Setting area and background

Nigeria—the most populous country in Africa with 140 million people [16]—is comprised of six geopolitical zones (GPZs), 36 states, and 774 Local Government Areas (LGAs). Northern Nigeria is historically underserved and suffers the highest infant mortality rate (IMR) and under five mortality rate (U5MR) [11]: the North East and North West GPZs have an IMR of 77 and 89 per 1,000 live births [11] and a U5MR of 160 and 185 per 1,000 live births [11], respectively. This is higher than the national IMR of 69 per 1,000 live births and U5MR of 128 per 1,000 live births [11].

In order to address these regional disparities, the Targeted States High Impact Project (TSHIP)—a five-year reproductive, maternal, newborn and child health and family planning (RMNCH/FP) program—was implemented in Bauchi, a state with a population of 4.6 million in the North East GPZ, and Sokoto, a state with a population of 3.7 million in the North West GPZ [16]. TSHIP—implemented by JSI Research and Training Institute, Inc. from 2009 through 2015—was the first United States Agency for International Development (USAID) full-state approach to providing RMNCH/FP services in Nigeria. This approach addressed the challenges of effective donor collaboration, program efficacy, and health systems strengthening. Malaria interventions, especially for pregnant women and children under five, were implemented in these states through TSHIP. Bauchi and Sokoto experience the highest transmission of malaria from April to October.

In order to contextualize malaria care-seeking, understanding the structure of the Nigerian health care system is necessary, as well. In the public sector there are dispensaries, primary health facilities (e.g., Primary Health Clinics [PHCs]), secondary facilities, and tertiary hospitals [8]. The private sector is made up of patent medicine vendors (PMVs), private clinics and hospitals. The Ministry of Health (MOH) is responsible for providing operational supervision to the private sector to ensure compliance standards. However, enforcing these standards is difficult due to inadequate numbers of qualified personnel to carry out this task [17].

Despite poor regulation and standards of care, the largest source of treatment for children (and adults) with fever is PMVs (45.6%) and the second largest are government health facilities (33.8%) [11]. This is alarming due to the fact that PMV staff lack formal training and supervision [17] and serve a large proportion of patients.

In addition to high mortality rates and poor service delivery, commodity management is also poor in this region. Drug supply management has been an on-going problem, especially at the PHC level [13]. A stock-out analysis of ACTs and RDTs done by TSHIP in Sokoto showed a deficit of 2 million ACT doses and 700 thousand RDTs to meet the current malaria burden [18]. Thus, data to help identify patterns in care-seeking will allow us to inform programmatic and policy activities to strengthen malaria case management and to better serve rural and underserved populations.

### Study design and implementation

**Figure 1:**
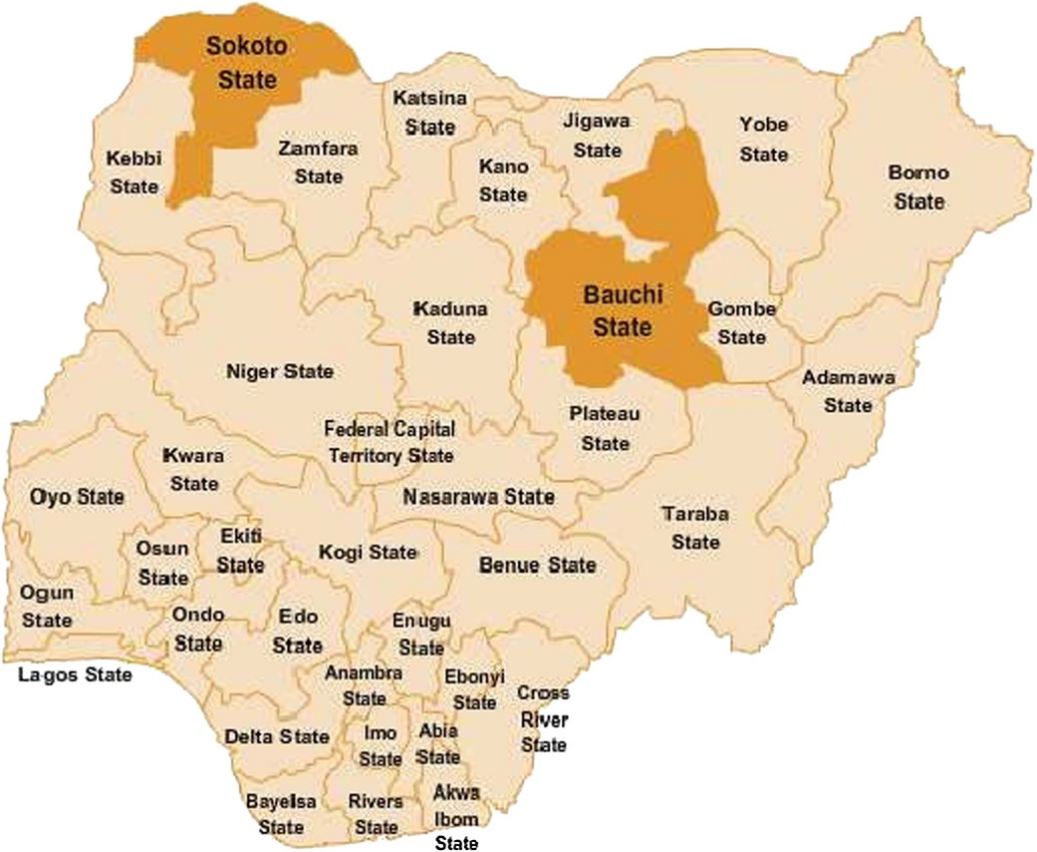
**Map of Nigeria: Bauchi and Sokoto highlighted.**

In November and December 2012, a relatively low period of malaria transmission, a cross-sectional lot quality assurance sampling (LQAS) survey was administered proportionally throughout each of the 20 LGAs in Bauchi and 23 LGAs in Sokoto (Figure 1 [19]). This was the baseline survey of an annual survey for monitoring and evaluation for TSHIP.

LQAS originated in the manufacturing sector to test the quality of a lot of goods. However, several studies and health programmes have used LQAS as a methodology to assess programme coverage and performance indicators in public health [5, 20]. This method of sampling divides a programme area into smaller geographic “lots,” or supervision areas, and then samples households from each lot. The results are then compared with a decision rule, or benchmark value, to help local TSHIP planners identify areas that are meeting or failing the benchmark value. This ability to prioritize the most vulnerable areas is crucial when resources are limited.

Nine separate questionnaires were implemented as part of the LQAS survey, which covered topics on maternal, newborn, and child health; malaria; and family planning to assess TSHIP’s programmatic activity. The data from “Questionnaire 8: Mothers of children 0 to 59 months with fever in the last 2 weeks” as well as “Questionnaire 1: Women of reproductive age with children aged 0 to 59 months” are used in this paper.

In Bauchi and Sokoto, each LGA acted as a lot or a supervision area. Within each lot, 19 settlements (villages) were randomly selected, proportional to population and representative of each LGA, to complete one comprehensive LQAS survey. LQAS methodology is based on a decision rule of choosing 12–30 settlements per lot. Nineteen settlements per lot have been proven to give a good fit for an annual coverage target of 50% or below with a sensitivity of 95% [21]. A household in each settlement was randomly selected and all applicable questionnaires were completed. If a selected household was not eligible to answer all nine questionnaires, the surveyor moved on to the next household to finish the remaining questionnaires. This approach, called “parallel sampling”, is unique to LQAS. If a settlement was too small to complete all nine questionnaires, a neighboring settlement was visited to complete the survey.

In a household where two or more eligible respondents were found, one respondent was randomly selected. Interviewers were trained on the LQAS methodology and use of the pre-tested tools over a period of five days. Questionnaire 1 contained detailed maternal demographic questions. The questionnaire (number 8) on malaria included questions about maternal and child’s age, whether treatment was sought outside of the home, promptness of treatment, location of treatment, whether the child received a diagnostic test (either microscopy or RDT), whether the child took a medication, and what type of medication the child took.

In this analysis, we combine all of the small samples to create an unbiased random sample of 814 households representing the states of Bauchi (n = 379) and Sokoto (n = 435). With this much larger sample size, there is more power to describe care-seeking and treatment patterns, and identify differences between the two states. Using the chi-square test, a 5% significance level, and the given sample sizes, there is 80–90% power to identify a 10% or greater difference between the states across the range of possible response percentages. Moreover, percentages reported for an individual state, or both states combined, have margins of error ranging from 2–5%.

### Participants

Both the first and malaria questionnaires included women of reproductive age (15–49 years old) with children aged 0–59 months. The malaria questionnaire only included children who had a fever in the last two weeks. Detailed descriptive and demographic information is reported from the first questionnaire, since it was not collected on the malaria questionnaire. Some, but not all, mothers completed both questionnaires. Since both questionnaires focused on the same target population in the same villages the demographics should represent the women who completed the malaria questionnaire. Maternal and child age, and child’s sex, which were ascertained on both questionnaires, were statistically similar between the two survey samples.

### Variables

Prompt treatment was defined as treatment on either the same or following day (24 hours) of fever onset based on recommendations from the RBM Partnership [6]. In addition, if the mother said the child received treatment, but indicated location of treatment as home and provider as self, the child was re-categorized as not receiving treatment, since treatment was not sought outside of the home.

Treatment location was categorized into formal and informal sectors. Formal treatment locations included health facilities and private doctors. Informal treatment locations included PMVs, community based health volunteers (CBHV), traditional practitioners, and other. Included in the informal “other” category are unauthorized drug sellers, friends/relatives, and other.

### Statistical methods

To assess the primary aim of defining current care-seeking and treatment patterns for children under five with fever, frequencies of survey question responses were computed. Responses were also cross-classified by state, and the chi-square test was used to assess the statistical significance of geographic differences. To address a secondary aim of comparing these results to the NMCP/WHO standard case management pathway, results were organized according to the standard and the number and percent of respondents meeting each component of the case management pathway was estimated. Epi Info version 7 [22] was used for data entry and data cleaning and Stata/IC 12.1 [23] was uses for data analysis.

To assess another secondary aim—identifying whether maternal age, child age and gender, and state help predict if a child under five with fever is taken to treatment—a binary logistic regression was performed with seeking treatment as the dependent variable. Akaike information criterion and likelihood ratio tests (LRTs) were used to assess the explanatory power of the model and identify factors that contributed significantly to each model.

### Ethical approval

Ethical approval was granted by the Sokoto and Bauchi State Health Research Ethics Committee and by local leaders in the study area. Approval from the National Health Research and Ethics Committee is not required when a study is done in less than three states. Participants in the survey gave written consent before answering the questionnaire.

## Results

### Respondent characteristics

Table 1 showcases the descriptive statistics of the study population. Stratification of the data by state shows significant demographic differences between states (p <0.05). Therefore, further analyses were done with all data combined and stratified by state. Of the 818 women and children pairs that answered this survey, four were excluded because they did not meet age criteria. Therefore, 814 woman and child pairs were included in the analysis.

**Table 1:** **Socio-demographic characteristics of mothers and children under five with fever**

	Bauchi (N = 379)		Sokoto (N = 435)		Total (N = 814)		P-value
	N	%	N	%	N	%	
**Mother’s characteristics**							
**Age**							
Mean age (SD)	25.2 (6.3)		26.9 (6.7)		26.1 (6.6)		**0.0002**
15–19 years	58	15.3%	59	13.6%	117	14.4%	**0.003**
20–24 years	114	30.1%	90	20.7%	204	25.1%	
25–29 years	93	24.5%	104	23.9%	197	24.2%	
30–34 years	73	19.3%	107	24.6%	180	22.1%	
35–49 years	41	10.8%	75	17.2%	116	14.2%	
**Education** ^*^							
No education	263	69.4%	387	89.0%	650	79.8%	**0.000**
Primary education	77	20.3%	27	6.2%	104	12.8%	
Secondary education	30	7.9%	8	1.8%	38	4.7%	
Higher education	9	2.4%	5	1.2%	14	1.7%	
**Religion**							
Muslim	352	92.9%	429	98.6%	781	96.0%	**0.000**
Catholic	3	0.8%	1	0.2%	4	0.5%	
Other christian	22	5.8%	2	0.5%	24	3.0%	
Traditionalist	2	0.5%	3	0.7%	5	0.6%	
**Marital status**							
Married	376	99.2%	422	97.0%	798	98.0%	**0.02**
Not-married	3	0.8%	13	3.0%	16	2.0%	
**Children’s characteristics**							
**Age**							
Mean (SD)	16.0 (12.3)		17.9 (13.0)		17.0 (12.7)		**0.03**
0–6 months	93	24.5%	92	21.2%	185	22.7%	**0.008**
7–12 months	87	23.0%	95	21.8%	182	22.4%	
13–18 months	79	20.8%	61	14.0%	140	17.2%	
19–24 months	57	15.0%	96	22.1%	153	18.8%	
25–59 months	63	16.6%	91	20.9%	154	18.9%	
**Sex**							0.7
Male	199	52.5%	234	53.8%	433	53.2%	
Female	180	47.5%	201	46.2%	381	46.8%	
**Household characteristics**							
Multifamily household	34	9.0%	95	21.8%	129	15.8%	**<0.0001**

Bold values are significant at a p <0.05 level.

^*^Eight missing values for education. 814 used as denominator (N = 806).

Mean maternal age was 26.1 (15–48) years. The vast majority of mothers had no education (79.8%, 650) and only a small proportion had completed some higher education (1.7%, 14). The proportion of uneducated women was significantly greater in Sokoto (89.0%, 387) than Bauchi (69.4%, 263) (p <0.001). Most mothers were Muslim (96%, 781) and married (98%, 798). Children’s ages ranged from 0–59 months with a mean age of 17.0 months. Of the children, 53.2% (433) were male and 46.8% (381) were female. In addition, 15.8% (129) of mother and child dyads were part of a multifamily household.

### Treatment seeking behaviour

Of children under five with fever in the past two weeks, 76.7% (624) were brought to treatment. Of those children who were brought to treatment outside of the home (624), 61.5% (384) followed WHO and NMCP standards to do so promptly, 46.0% (287) received treatment in the formal sector and 54.0% (337) received treatment in the informal sector. These behaviours did not differ by state.

Treatment was most often sought at PMVs (45.5%, 284), followed by health facilities (43.8%, 273). The remainder sought care at CBHVs, private doctors, traditional practitioners, and other sources (3% or less of the time, respectively) (Tables 2 & 3). Treatment location differed by state only for CBHVs, with children accessing them 4.6% (15) of the time in Sokoto and 1.7% (5) of the time (p = 0.04) in Bauchi (Table 2).

**Table 2:** **Care-seeking and treatment patterns for children under five with fever**

	Bauchi (N = 379)		Sokoto (N = 435)		Total (N = 814)		P-value
	N	%	N	%	N	%	
**Sought Treatment**							
Yes	299	78.9%	325	74.7%	624	76.7%	0.16
No	80	21.1%	110	25.3%	190	23.3%	
	**Bauchi (N = 299)**		**Sokoto (N = 325)**		**Total (N = 624)**		
**Location of first-line treatment or advice sought**							
**Formal**	146	48.8%	141	43.4%	287	46.0%	0.17
Health facility	138	46.2%	135	41.5%	273	43.8%	0.24
Private doctor	8	2.7%	6	1.8%	14	2.2%	0.45
**Informal**	153	51.2%	184	56.6%	337	54.0%	0.17
PMVs	136	45.5%	148	45.5%	284	45.5%	1.00
CBHV	5	1.7%	15	4.6%	20	3.2%	**0.04**
Traditional Practitioner	5	1.7%	10	3.1%	15	2.4%	0.26
Other^i^	7	2.3%	11	3.4%	18	2.9%	0.41
**Timing of first-line treatment**							
Prompt	192	64.2%	192	59.1%	384	61.5%	0.19
Not Prompt	107	35.8%	133	40.9%	240	38.5%	
**Treatment**							
Received diagnostic blood test	30	10.0%	31	9.8%	61	9.8%	0.84
Received medication	245	81.9%	237	72.9%	482	77.2%	**0.02**
Received non-anti-malarial medication	131	46.4%	102	35.3%	233	37.3%	0.38
Received anti-malarial	114	38.1%	135	41.5%	249	39.9%	0.38
Received prompt anti-malarial	73	24.4%	92	28.3%	165	26.4%	0.27
Received non-ACT anti-malarial	79	28.0%	107	37.0%	186	29.8%	**0.004**
Received prompt non-ACT anti-malarial	51	18.1%	69	21.2%	120	19.2%	**0.01**
Received ACT	35	11.7%	28	8.6%	63	10.1%	0.20
Received prompt ACT	22	7.4%	23	7.1%	45	7.2%	0.89

Bold values are significant at a p <0.05 level.

^i^Friend/Relative/Unauthorized Drug Seller/Other.

**Table 3:** **Care-seeking patterns and treatment by location**

	Prompt treatment	Test done	Anti-malarial given	ACT given	Other medication given
**Health facility (273)**	58.6% (160)	15.0% (41)	46.5% (127)	14.3% (39)	29.3% (80)
**Private doctor (14)**	71.4% (10)	14.3% (2)	35.7% (5)	14.3% (2)	42.9% (6)
**PMV (284)**	64.4% (183)	6.0% (17)	38.0% (108)	7.0% (20)	40.8% (116)
**CBHV (20)**	45.0% (9)	0%	25% (5)	10.0% (2)	45% (9)
**Traditional practitioner (15)**	73.3% (11)	6.7% (1)	13.3% (2)	0%	66.7% (10)
**Other (18)**	61.1% (11)	0%	11.1% (2)	0%	66.7% (12)

### Testing

Of children who sought treatment, only 9.8% (61) received a blood test (Table 2). Children were most likely to receive a blood test if they received treatment at a health facility (15.0%, 41), next at a private doctor (14.3%, 2), traditional practitioner (6.7%, 1), and, last, a PMV (6.0%, 17) (Table 3).

### Medications received

Of children who received treatment, 39.9% (249) received an anti-malarial, however 29.8% (186) received a non-ACT anti-malarial and only 26.4% (165) received a prompt anti-malarial (Table 2). A child was most likely to receive an anti-malarial if they went to a health facility (46.5%, 127), next at a PMV (38%, 108), private doctor (35.7%, 5), CBHV (25%, 5), and traditional practitioner (13.3%, 2) (Table 3).

**Figure 2:**
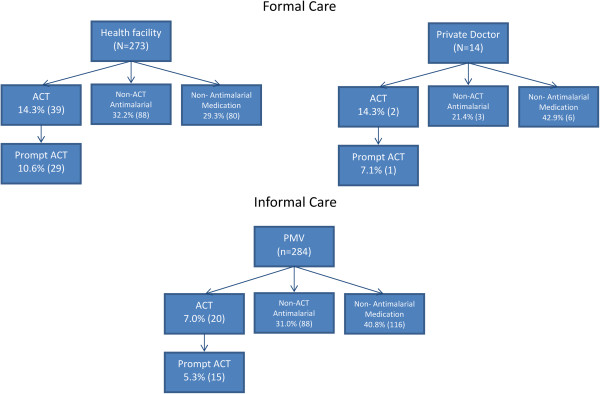
**Medication type given by treatment location to children under five with fever.** Percentages in boxes are proportions of children who sought care at that location.

An even smaller proportion of children received a WHO and NCMP recommended ACT (10.1%, 63), and only 7.2% (45) received a prompt ACT (Table 2). Children were most likely to receive an ACT (14.3%) if they received treatment at a health facility (39) or private doctor (2), next from a CBHV (10.0%, 2) and then a PMV (7.0%, 20) (Table 3, Figure 2). Children were most likely to receive a prompt ACT if they received treatment at a health facility (10.6%, 29), next a private doctor (7.1%, 1), and last a PMV (5.3%, 15) (Figure 2). Treatment results did not differ by child’s sex (p ≥0.17), but did occasionally differ by state (p <0.02) (Table 2).

### Treatment based on national policy/WHO recommended pathway

**Figure 3:**
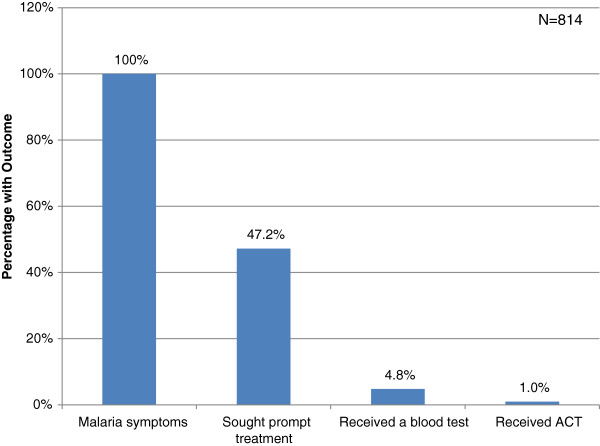
**Treatment pathway for children under five with fever who received NMCP/WHO standard care.**

Figure 3 illustrates the NMCP/WHO standard treatment pathway. Of children under five who had a fever in the last two weeks (814), 47.2% (384) sought prompt treatment; of those that sought prompt treatment, 4.8% (39) of the total sample received a blood test; of those that received a blood test, 1.0% (8) of the total sample received an ACT.

**Figure 4:**
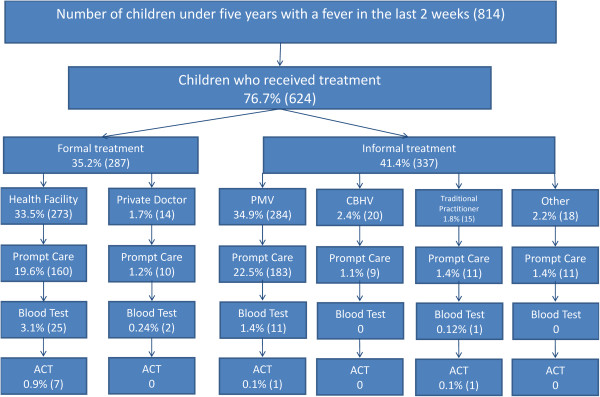
**Treatment pathway for children under five with fever who received NMCP/WHO standard care, by location.** Percentages in boxes are proportions of all febrile children.

In addition, Figure 4 illustrates which proportion of children received the NMCP/WHO standard of care stratified by treatment location. Of all locations, health facilities provided the greatest percentage of NMCP/WHO standard care (0.9%, 7). PMVs and traditional practitioners each treated one patient according to the NMCP/WHO standard, accounting for 0.1% of children with fever, respectively.

### Predictors of treatment seeking

Table 4 presents the results of the logistic regression created to identify factors that help predict whether a child received treatment outside of the home. The results show that neither state, mother’s age, nor child’s sex was significantly related to treatment seeking. Child’s age was somewhat predictive of treatment seeking, but the pattern was not strictly linear. The clearest indication from the data was that treatment seeking was less likely for children under the age of six months. There was a significantly greater odds of seeking treatment for 7–59 month olds (OR: 1.74, p = 0.003) compared to 0–6 month olds, adjusted for mother’s age, child’s sex, and state.

**Table 4:** **Multivariable logistic regression models of the odds of seeking any treatment for a child under five years with fever from any location**

	Unadjusted (OR)			Adjusted (OR)		
	Total			Total		
Variable	OR	95% CI	P value	OR	95% CI	P value
**Mother’s age**	1.00	0.98, 1.03	0.33	1.00	0.98, 1.03	0.84
**Child’s age**						
0–6 months	1.0			1.0		
7–59 months	1.72	1.19, 2.48	**0.004**	1.74	1.16, 3.03	**0.003**
**Childs sex**						
Male	1.0			1.0		
Female	0.87	0.63, 1.20	0.40	0.86	0.62, 1.20	0.38
**State**						
Bauchi	1.0			1.0		
Sokoto	0.79	0.57, 1.10	0.16	0.77	0.55, 1.07	0.12

Bold values are significant at a p <0.05 level.

## Discussion

### Care-seeking

Rates of treatment, prompt treatment, and formal sector treatment in Northern Nigeria are 77%, 47%, and 35% respectively (N = 814). This rate of treatment seeking is consistent with a 2013 study in Nigeria in which 70% of parents of children under five sought advice or treatment [11]. These rates are also slightly higher than rates discovered in a similar study of malaria treatment in Senegal where 62% of children under five with fever in the last two weeks received treatment, 40% received prompt treatment, and 32% received treatment from the formal sector [15]. However, there is much room for improvement since the malaria treatment seeking rates in Benin, DRC, Madagascar, Uganda, and Zambia are significantly higher (83 – 95%) [22]. Since only 47% of caregivers sought prompt care and 35% sought formal care, future programmatic emphasis may be placed on educating caregivers *where* and *how quickly* they need to seek treatment for children under five with fever. Future research in this area may also focus on assessing recognition of malaria symptoms and knowledge of case management strategies among families with children under five years old.

### Location of care

PMVs were found to be the preferred treatment providers, with 46% of all children under five with fever receiving treatment from this location. This is consistent with the national rate of 46% [11] and other areas such as the Kabale district in Uganda (53%) [23]. One reason for this may be that families feel that local private providers are more accessible, are more sensitive to the patients’ needs, and spend more time with them than public sector providers [24, 25]. However, there are key problems with this sector including inappropriate storage [25], knowledge, and dosing of drugs [24, 26].

Another downfall of seeking treatment at a PMV is that they are not authorized to administer diagnostic tests [personal communication, Dr. Mohammed Ibrahim]. This was evident by a recent survey that found microscopy completely unavailable at PMVs and RDTs available at 1% of PMVs [27]. In order to promote all steps in the malaria case management pathway, policy recommendations are to facilitate stocking of and training of staff in the use of RDTs at PMVs. In addition, when RDTs were implemented in the private sector, providers were not knowledgeable in treatment for non-malaria fever, which resulted in treatment with an ACT despite a negative RDT [28]. Therefore, capacity development and quality assurance of this sector is an important piece to improving malaria case management.

Less than half (44%) of the parents sought treatment for their children from a health facility, even though health facilities provided the largest proportion of children with recommended case management (Table 3). This rate is actually higher than the national rate of children receiving care at a public facility (35%) [11].

### Use of diagnostic tests

Despite a national benchmark to promptly test 80% of children under five with fever [8], in this study, children rarely received a blood test (8% of sample, 10% of care-seeking children). This is lower than the national rate of 11% [11], and much less than the average rate across 13 sub-Saharan African countries (17%) [29] and Rwanda (30%) [30], but higher than the rate of testing in Sierra Leone (1%) [31].

Although health facilities tested the largest proportion of children in this study (15%), that proportion is far below the testing rate of 95% in health facilities in Zambia [32]. In order to bridge the current gap between NMCP/WHO standard and current treatment, policies and programmes may focus on making blood tests and ACT more consistently available. A survey done by ACTwatch throughout Nigeria in 2011 showed that microscopic testing was more available than RDTs [27], therefore policies and initiatives to improve diagnostic testing should be consistent with commodity availability and acceptability. More specifically, of locations that stocked anti-malarials in the last three months, only 26% of public health facilities, 37% of private for profit facilities, and 1% of PMVs offered any testing services [27]. These stock-outs are a key weakness is the ability of facilities to provide diagnostic blood testing consistent with WHO/NMCP standards for malaria case management.

### Treatment given

Of anti-malarials given, 75% were non-ACT, much higher than the national average of 26% [11] This is of great concern because malaria is resistant to many anti-malarials in Nigeria [8] and ACT has been the standard of care since 2005 [6]. The rate of standard treatment using prompt ACT is 7%; higher than the national rate of 4%, but much lower than a study in Tanzania (38%) [33]. It is likely that a combination of factors influence the low ACT rate. For example, a 2008 study showed only 36% of PHC health workers in Sokoto had adequate knowledge of malaria case management [34]. In addition, another study showed that only 49% of public facilities in Nigeria have at least one quality assured first-line dose of ACT [35].

Given the fact that PMVs are the most commonly accessed treatment location, interventions to strengthen their role in malaria case management may result in improved outcomes. In Kenya, an intervention that subsidized ACT for retail outlets, trained retail outlet workers, and strengthened community awareness of case management standards, saw a 25% increase in ACT treatment for children under five. In addition, a USAID report recommends engaging the private and even informal sector to improve coverage and case management of child illness [24]. Policy and logistics planning should address the lack of training and shortages of essential malaria commodities in recommended and frequently accessed treatment locations.

Given the current stock-outs of ACT and RDTs [18] in Sokoto State, commodity procurement and logistics need to be improved to bridge the gap between available and needed RDTs and ACT. Further research may be needed to understand weaknesses in the supply chain and training of health professionals. Future programmatic and policy activity should focus on strengthening these aspects of health care infrastructure.

### Overall and NMCP/WHO standard treatment patterns

While the majority of caregivers (77%) seek treatment for children under five presenting with fever, when care-seeking pathways are studied, only 1% of children under five with fever receive treatment according to WHO and Nigeria’s NMCP standards: prompt care-seeking, diagnosis with an RDT, and treatment with an ACT. This leaves a staggering discrepancy between the Nigeria NMCP and RBM goal coverage rate of 80% for prompt RDT use and subsequent ACT treatment if indicated [4, 6, 8] and the actual rate among children under five with fever in Northern Nigeria (Figure 3).

Policy and caregiver education strategies are important, but will have limited effect on improving malaria case management if health systems remain weak. In Nigeria, current health system weaknesses include stock-outs due to poor procurement and supply chains for malaria commodities in the public health sector, weak delivery of health services in public facilities, lack of necessary and adequately-trained human resources for health, and nearly non-existent diagnostics [4].

The poor state of malaria care in Sokoto and Bauchi could be improved by strengthening national policy and regulatory power in the informal sector. Since informal sector regulation and systems strengthening is often a difficult a lengthy process, incentives such as vouchers or conditional cash transfers may be used to direct patients toward the formal sector until the private and informal sector is can correctly deliver malaria case management.

### Predictors of treatment

Child’s age was a significant predictor in the regression for seeking treatment versus not seeking treatment. However, our modeling was limited by the number of variables on the malaria questionnaire. Thus, other factors may be as, or more, predictive of seeking treatment. However, cultural practice could plausibly contribute to the age differences. Several studies have shown that physical confinement is common in Nigerian postpartum practices—often lasting 40 days [36–38]. Confinement has implications for the ability of the mother to seek care for her febrile newborn and may have affected the decreased odds of seeking care for 0–6 months. However, this information may be inconclusive due to the limitations in variables and sample size. Further research should be done to assess factors that facilitate recommended malaria case management.

### Limitations

There are some limitations to using a study based on LQAS methodology to assess care-seeking behaviour of parents. LQAS methodology is designed to cost-effectively compare programme coverage rates with set thresholds to evaluate programme performance. Thus, LQAS surveys typically have relatively small sample sizes (low power) and a relatively limited number of questions. This makes it difficult to perform more complex statistical analyses to establish causal and associative relationships. However, with over 800 respondents, there was good power to assess care-seeking, with a margin of error of only 2–5% for each question, but a limited number of explanatory variables.

Similarly, although polygamy is common in the study area, the survey did not assess whether each household was comprised of a polygamous or monogamous family; a study in Ethiopia found that mothers in a monogamous marriage had 3.4 greater odds of seeking treatment for a child under five with fever than mothers in a polygamous marriage [39]. The survey did not specifically ask if medications were procured at location of first line treatment or elsewhere. Thus, it is assumed that the medication was obtained from the first place of treatment. Lastly, the study did not assess the result of the diagnostic blood test. This is a limitation to assessing quality of treatment, since the proportion who received a medication cannot be compared to those who were diagnosed with malaria. However, malaria prevalence rates are so high in these areas, it is assumed the majority of fever cases were malaria.

Several types of bias may have been introduced in the study, but should not affect the conclusions of the analysis. Recall bias is a risk given this was a retrospective cross-sectional survey, compounded by the fact that largely uneducated women were asked to recall complex information. However, this bias should be minimal since the maximum time that had to be recalled was two weeks. Misclassification bias was minimized by only classifying a child as receiving treatment if the mother answered both yes to the child receiving a medication and listed what the medication was, even if it was “other” or “unknown”.

## Conclusions

Nigeria’s NMCP goal coverage rates of 80% for NMCP and WHO standard malaria case management for children under five with fever in Northern Nigeria are far from being met. Although the best care is provided in health facilities, children are most likely to visit a PMV for first-line treatment of fever. The implementation of national policy change is stunted by drug stock-outs, inadequately trained service providers, and caregivers of children under five with a lack of knowledge of correct malaria treatment. Given the ability to treat a child with malaria differs greatly between facility types, policy and logistics planning should address the shortages of essential malaria commodities in recommended and frequently accessed treatment locations. Particular emphasis should be placed on integrating the private sector into standardized care and educating caregivers on the necessity for testing before treatment and availability of free ACT in public health facilities for the treatment of uncomplicated malaria.

## Abbreviations

ACT: Artemisinin-based combination therapy
AMFm-ACT: Affordable medicine facility-malaria artemisinin combination therapies
CBHV: Community- based health volunteer
FMOH: Federal ministry of health
GPZ: Geopolitical zone
IMR: Infant mortality rate
ITN: Insecticide-treated bed net
LGA: Local Government Area
LQAS: Lot quality assurance sampling
LRT: Likelihood ratio test
MIS: Malaria indicator survey
MOH: Ministry of health
NMCP: National malaria control programme
OR: Odds ratio
PHC: Primary health clinic
PMV: Patent medicine vendor
RBM: Roll back malaria partnership
RDT: Rapid diagnostic test
RMC: Role model care givers
RMNCH/FP: Reproductive maternal, newborn and child health and family planning
SES: Socio-economic status
TSHIP: Targeted states high impact project
USAID: United States agency for international development
WHO: World Health Organization.
